# Measurement of knee joint space width with bi-planar radiography

**DOI:** 10.1093/jbmrpl/ziaf196

**Published:** 2025-12-27

**Authors:** Isabella D Vandergaag, Richard E A Walker, Steven K Boyd

**Affiliations:** McCaig Institute for Bone and Joint Health, Cumming School of Medicine, University of Calgary, Calgary, AB T2N 4Z6, Canada; McCaig Institute for Bone and Joint Health, Cumming School of Medicine, University of Calgary, Calgary, AB T2N 4Z6, Canada; Department of Radiology, Cumming School of Medicine, University of Calgary, Calgary, AB T2N 4Z6, Canada; McCaig Institute for Bone and Joint Health, Cumming School of Medicine, University of Calgary, Calgary, AB T2N 4Z6, Canada; Department of Radiology, Cumming School of Medicine, University of Calgary, Calgary, AB T2N 4Z6, Canada

**Keywords:** bi-planar radiography, X-ray, knee, joint space, reproducibility, osteoarthritis, low radiation

## Abstract

The objective of this study was to identify whether the joint space width (JSW) of the knee, measured by bi-planar imaging, is reproducible compared to the clinical reference radiography. Our cross-sectional study design included a cohort of uninjured individuals (*N* = 30, 26.7 ± 5.1 yr) who underwent scanning to determine the short-term precision of the technique, involving repeat scans by bi-planar radiograph. Additionally, repeat conventional tunnel view knee radiographs were used as a comparator. The minimum apparent tibiofemoral JSW was collected for each leg side and compartment for both modalities. The root-mean-square coefficient of variation (RMSCV) and least significant change (LSC) for bi-planar scans (RMSCV = 6.46%, LSC = 1.07 mm) were comparable to conventional radiography (CR) (RMSCV = 7.66%, LSC = 1.15 mm). There was a bias for greater JSW by bi-planar radiography than CR (9.0%, *p* < .01), particularly for the forward unloaded left leg lateral (15.5%) and medial (17.6%) compartments. In conclusion, we found that JSW measurements from bi-planar scanners are reproducible and comparable to CR. While radiography remains accessible clinically, accurate and precise JSW by bi-planar scanners is feasible provided knee position and alignment are controlled.

## Graphical Abstract

**Graphical Abstract:**
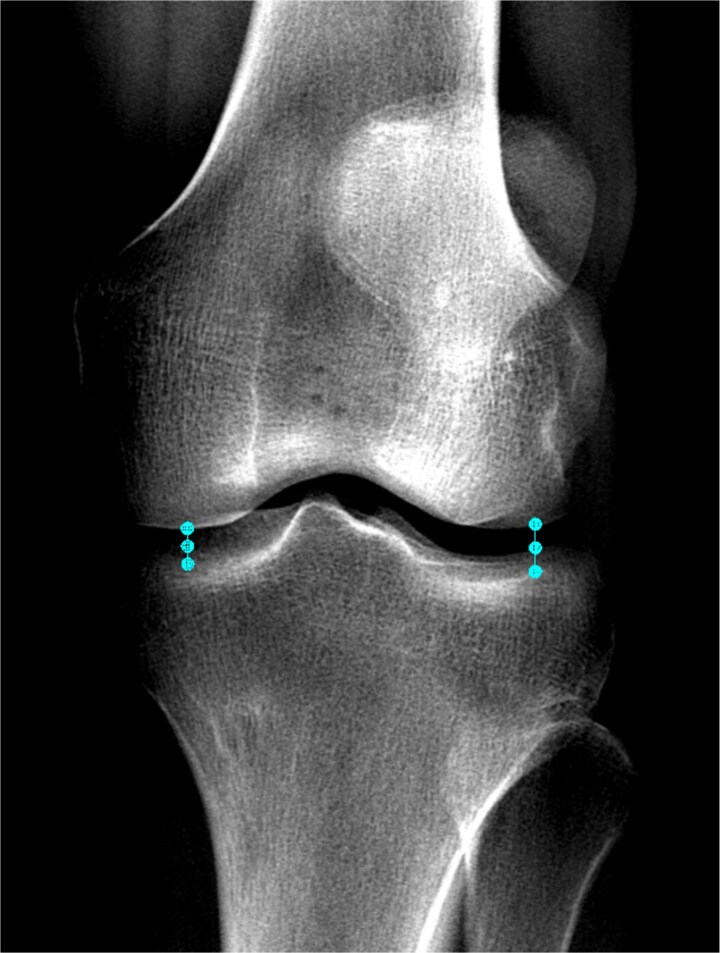

## Introduction

Lower limb imaging devices for assessing joint space width (JSW) and limb alignment are valuable for patients affected by anatomic variation, aging, and acute knee injuries that can predispose to osteoarthritis (OA).^1^^,^^2^ Bi-planar radiolography (EOS Imaging) offers simultaneous anteroposterior (AP) and lateral (LAT) projections of both lower extremities with low radiation exposure.^3^ Compared to conventional knee radiographs, bi-planar radiography has been reported to have approximately a 50% reduction in the dose-area product when employing conventional speed mode.^4^^,^^5^ Compared to a full-length lower extremity CT scout scanogram, the conventional-mode bi-planar scan (referred to as EOS-slow mode) has a higher radiation dose. However, employing a higher speed scan mode (referred to as EOS-fast; not used in this study), there is 20% of the skin entrance radiation dose when compared to CT. Future work could evaluate whether JSW measurement can be applied to EOS-fast mode acquisition, which would result in a lower dose by bi-plane radiography, although effects reproducibility are not clear. EOS addresses a notable gap in current methodologies, where conventional radiographs, full-length bilateral AP hip-to-ankle radiographs, and CT scout scanograms are limited due to small scan regions, higher radiation doses, and lack of weight-bearing capabilities, respectively.

Conventional radiographs are the clinical reference for assessing JSW for knee OA based on a single value of minimum JSW within each the medial and lateral tibiofemoral compartment. The minimum apparent JSW indirectly evaluates cartilage loss associated with degenerative joint disease.^6^ A single outcome measure may result in challenges in reproducibility as minimum JSW can vary due to repositioning differences between scans. The advent of 3D visualization modalities, such as MRI and CT, have the potential for comprehensive and precise JSW assessments by offering a full 3D assessment of the joint. A limitation of these modalities, however, is that measurements are performed non-weight-bearing. Additionally, three-dimensional techniques, such as CT, result in higher radiation compared to conventional radiographs. Another consideration might be cone-beam weight-bearing CT, which is a relatively new low dose modality. Scans can be performed in a fixed flexion position to evaluate JSW, representing an additional complementary 3D modality in this area.^7^^,^^8^ Overall, given that knee JSW measurements are load-dependent and an important metric for assessing OA, there is an opportunity to introduce alternative methods for monitoring JSW to evaluate tibiofemoral health and disease.

Bi-planar radiography is designed to provide several 2D alignment parameters, including leg length, knee valgus and varus alignment angle, hip-knee-shaft angle, femoral version, and knee flexion angle.^3^ There is a paucity of studies that have measured JSW by bi-planar radiography at the hip and knee in OA populations,^1^^,^^9^ and the reproducibility for measurements of JSW are not known.

The objective of this study is to assess bi-planar radiography for measuring JSW at the knee with direct comparison to conventional radiography (CR), which serves as the clinical reference. We hypothesize that bi-planar radiography offers a reproducible measure of knee JSW as determined by short-term repeat scans compared to CR. We report the accuracy, precision, and feasibility of bi-planar radiography for assessing knee JSW.

## Materials and methods

### Participants

A cohort of 30 healthy participants was recruited for our cross-sectional study and used to validate the reproducibility and accuracy of EOS lower extremity bi-planar radiography for JSW measurements compared to conventional posteroanterior (PA) semi-flexed conventional knee radiographs. Inclusion criteria were males and females above 18 yr of age, and a sex-balanced recruitment approach was employed. Exclusion criteria included patients with prior knee ligament tear (complete or partial), prior intra-articular fracture, and a history of knee surgery. For safety and data quality, pregnant women or women planning pregnancy within a year were ineligible. Finally, individuals with a history of disease or treatment affecting bone turnover in the past 12 mo (eg, prolonged corticosteroid use) were excluded. All participants completed repeat biplanar lower extremity radiography and repeat bilateral PA semi-flexed knee radiograph within a 1-h period by a certified medical radiation technologist.

### Medical imaging

**Figure 1:**
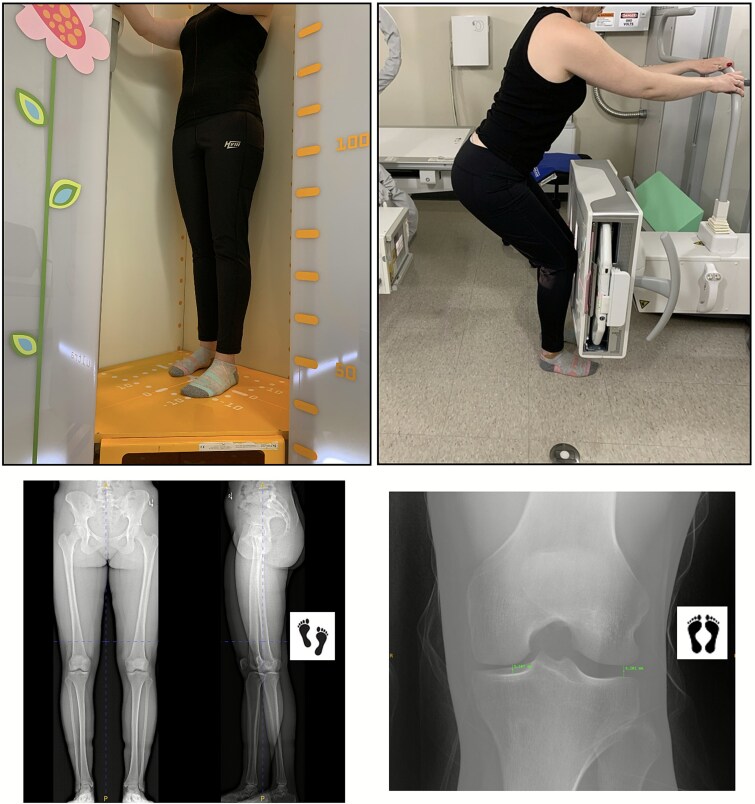
Anterior offset left foot placement for bi-planar lower limb radiographs (EOS Imaging) shown on left, with images below. Conventional radiography had both feet positioned side by side, with a resulting example image shown below.

Standard scanning protocols described by the manufacturer were used for bi-planar radiography (EOS Imaging; Plane A: 250 mA, kVp 85, focal spot 0.7 mm; Plane B: 320 mA, kVp 110, focal spot 1.3 mm; FOV top of pelvis to toes) with weightbearing AP and LAT projections of both lower limbs acquired simultaneously from the LS to the plantar aspect of the feet, a maximum craniocaudal distance of 175 cm.^4^ Participants were positioned with their feet in an anterior offset position with the forward unloaded left leg to distinguish the anatomical structures of the lower extremities on the lateral projection (Figure 1).^3^ The right foot was situated next to the first metatarsal bone of the left foot and a simple positioning aid (eg, a “foot map”) was used to measure foot offset and spacing and to assist repositioning for the repeat scan. A future approach might consider permanent offset marker lines on the floor (ie, similar to airport security) for toe alignment. Regardless of the approach, this standard offset positioning affords differentiation of the right and left leg in the lateral projection and permits measurement of knee flexion angle/recurvatum and femoral version. For conventional radiographs, bilateral PA digital image (Discovery XR656, GE Healthcare) of the knee was obtained in a semi-flexed position in a tunnel view configuration.^10^^,^^11^ Both legs were aligned side by side and feet placed straight and toes pointing forward. We did not use a flexion device^10^ or adjust for tibial slope using fluoroscopy (Figure 1).

Repositioning for biplanar imaging involved patients exiting the scanner, walking a few steps, then re-entering and placing their feet in the same position and allowing the technician to align the hips prior to scanning. Repositioning for CR involved a similar process of exiting and re-entering the X-ray.

### JSW analysis

Minimum JSW was defined at the perceived narrowest point in the medial and lateral tibiofemoral joint compartments.^12^^,^^13^ The JSW was measured using 2 margins: the distal convex margin of the femoral condyle and the proximal margin for the tibial articular surface.^14^ The tibial line, which represents the proximal margin, is defined by a superior margin (bright radio-dense band) of the subchondral cortex.^15^ These margins provided a conventionalized and reproducible method of measuring JSW. All JSW measurements were conducted by a single rater and intra-rater repeatability was determined. An open-source medical imaging software (ITKSnap v4.0.0 Kitware) was utilized to measure minimum JSW on the digital knee radiographs by a single rater (IV) and consistent voxel sizes ensured equal magnification. The bi-planar scanner built-in software was used to measure the equivalent distances between femoral and tibial bone landmarks to obtain minimum JSW (SterEOS 3.2.20).^3^^,^^16^

### Statistical analysis

The sample size (*N* = 30) and number of measurements (*n* = 2) yielded an upper 90% (or one-sided 95%) CI of less than +30%, sufficient for characterizing the precision of each procedure.^17^ Within-scanner precision was performed for both modalities. Precision was assessed by calculating the root-mean-square coefficient of variation (RMSCV) and the least significant change (LSC) between repeat measurements and repeat scans of bi-planar radiography and CR. The Shapiro–Wilk test was used for non-parametric data, and Wilcoxon paired and unpaired tests (significance threshold, *p* < .05) were conducted to compare JSW within subgroups of knee compartment, leg side, and sex. Bland–Altman (B-A) plots were created for all variables and calculated within the 95% limits of agreement. Percent difference for bi-planar and CR JSW was expressed as:


$$ \%\mathrm{Diff}=\frac{\left[\mathrm{bi}-\mathrm{planar}\ \mathrm{JSW}\right]-\left[\mathrm{CR}\ \mathrm{JSW}\right]}{\left[\mathrm{CR}\ \mathrm{JSW}\right]}\ast 100\%. $$

Accuracy was determined by linear regressions that established the slope and intercept for the relationship between bi-planar radiography and clinical reference CR. The coefficients of determination (R^2^) for all outcomes were calculated.

## Results

### Demographics for participants

The demographic characteristics of the cohort are presented in Table 1. The mean age of the participants was 26.7 yr (minimum of 20.8; maximum of 43.9 yr of age). The mean positioning parameters for bi-planar X-ray included the following: anterior offset (defined as distance between the distal margin of the left and right big toe (in cm)) of 8.7 cm and the horizontal toe distance (defined as the distance between the left and right hallux (in cm)) of 6.0 cm, and the horizontal heel distance (defined as distance between the left and right heel centers (in cm)) of 13.7 cm. All participants had knee alignment ±5° based on EOS scans.

**Table 1:** Demographics (*n* = 30; 50% female).

Descriptive measures	Mean	SD	Range	
			Minimum	Maximum
**Age (yr)**	26.7	5.1	20.8	43.9
**Height (cm)**	173.1	11.3	156.0	204.0
**Weight (kg)**	73.6	12.0	53.7	106.0
**BMI (kg/m** ^**2**^**)**	24.5	2.6	19.9	30.5
^**a**^ **Anterior offset (cm)**	8.7	2.2	4.7	12.7
^**b**^ **Horizontal toe distance (cm)**	6.0	2.1	1.2	8.6
^**c**^ **Horizontal heel distance (cm)**	13.7	3.4	1.3	19.0

Continuous variables are given as mean, SD, minimum, and maximum.

a defined as distance between the most distal margin of the right and left great toe (in cm).

b defined as the distance between the left and right hallux (in cm).

c defined as distance between the left and right heel centers (in cm).

### Within scanner precision

No significant difference was detected between the precision of bi-planar radiography (RMSCV = 6.46, LSC = 1.07 mm) and CR (RMSCV = 7.66, LSC = 1.15 mm) (Table 2). Additionally, the intraclass correlation (ICC) value for the repeated bi-planar scans (0.901) and the CR scans (0.864) reflects good agreement of measurements between scans for both modalities. Intra-rater repeatability assessed on a randomized subset was assessed (ICC = 0.916; 95% CI: 0.88-0.94).

**Table 2:** Joint space width reproducibility by modality and tibiofemoral compartment (based on the root-mean-square coefficient of variation (RMSCV) and least significant change (LSC)).

Participants (*n* = 30)	Bi-planar radiography		CR	
	RMSCV (%)	LSC (mm)	RMSCV (%)	LSC (mm)
**Left lateral**	7.75	1.45	9.74	1.64
**Left medial**	7.55	1.17	7.83	1.05
**Right lateral**	5.75	1.02	7.21	1.13
**Right medial**	4.80	0.65	5.84	0.77

### Between-modality agreement

**Figure 2:**
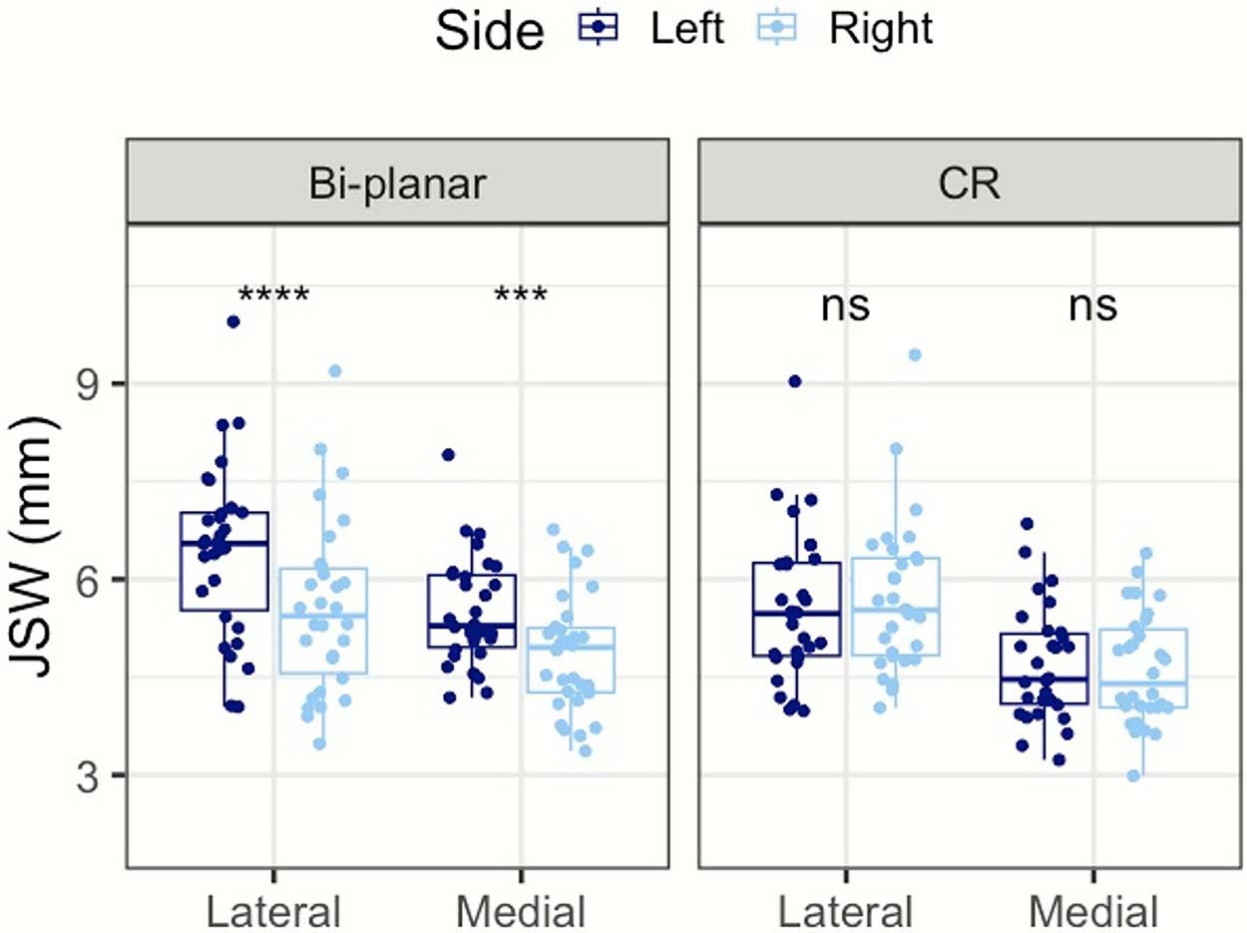
Boxplot showing the minimum joint space width (JSW) measurements between bi-planar radiography and conventional radiography (CR). The lateral (navy) and medial (blue) compartments of the knee are shown. Boxplots show the first quartile (25th percentile) and the third quartile (75th percentile) with the median line (50th percentile). Significance is indicated with ^*^^*^^*^^*^p < .0001 and ^*^^*^^*^p < .001.

The minimum JSW are reported for biplanar radiography and CR modalities by tibiofemoral compartment (Table 3). A significant difference between the modalities was found in the forward left knee for both the lateral (*p* < .01, 15.5%) and medial (*p* < .001, 17.6%) tibiofemoral compartments, with both compartments consistently measuring a greater minimum JSW on bi-planar radiography (Figure 2). However, the mean difference between modalities were not statistically significant for the right knee (back positioned leg) for both the lateral (*p* = .09) and medial (*p* = .15) tibiofemoral compartments.

**Table 3:** Joint space width (JSW) by modality and tibiofemoral compartment.

JSW measures (mm)	Bi-planar radiography			CR			*p* value	Percent difference %
	Mean	Range		Mean	Range			
		Min	Max		Min	Max		
**Left lateral**	6.4 ± 1.3	4.0	10.0	5.6 ± 1.1	4.0	9.0	.003	15.5
**Left medial**	5.5 ± 0.8	4.2	7.9	4.7 ± 0.9	3.2	6.9	<.001	17.6
**Right lateral**	5.6 ± 1.3	3.5	9.2	5.7 ± 1.2	4.0	9.4	.09	−2.7
**Right medial**	4.9 ± 0.9	3.4	6.8	4.6 ± 0.9	3.0	6.4	.15	5.4

Significance is shown for the between-modality analysis for each parameter.

**Figure 3:**
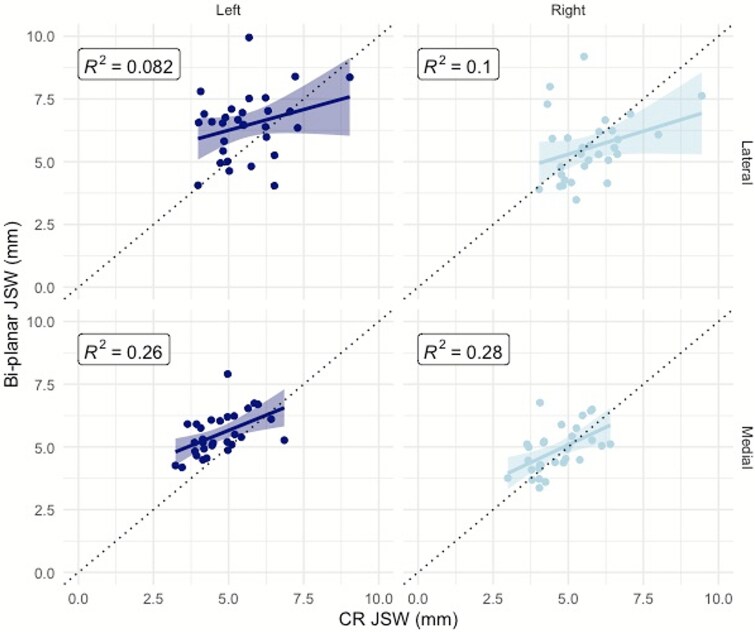
Regressions for comparisons of minimum joint space width (JSW) measurements assessed using bi-planar radiography and conventional radiography (CR). Data is shown for the lateral compartment (top) and medial compartment (bottom) of the left (navy) and right (blue) knees. The dashed black line is the y~x plot and the solid colored line is the linear regression.

**Figure 4:**
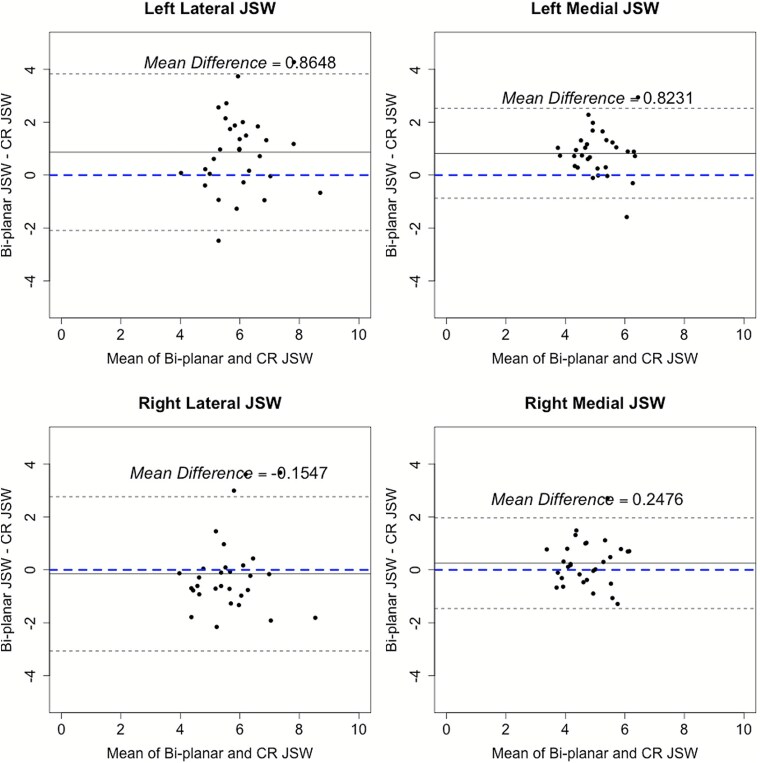
Comparison of minimum joint space width by Bland–Altman analysis showing mean difference (solid gray line) and 95% limits of agreement (dotted gray lines). Joint space width between modalities for left forward leg (top) and right back leg (bottom) for both the lateral and medial tibiofemoral compartments. The dashed blue line is the plot of x = 0.

Linear regressions (Figure 3) and Bland–Altman plots (Figure 4) provide a comparison of the 2 modalities. The linear regression indicated weak correlations between scanners. The best agreement (R^2^ = 0.28) was observed in the right medial compartment, whereas the forward left leg lateral compartment had the weakest agreement (R^2^ = 0.082). Results of the B-A demonstrated that JSW measurements obtained by bi-planar radiography are systematically greater than those measured by CR.

## Discussion

This study demonstrated that both bi-planar and conventional PA semiflexed knee radiographic techniques provide good reproducibility of minimum JSW measurements for the medial and lateral tibiofemoral compartments (ICC of 0.901 and 0.864, respectively). However, direct comparison between modalities revealed statistically significant systematic differences in the minimum JSW measurement of both the medial and lateral compartments (*p* < .01), with generally a larger minimum JSW measured with bi-planar radiography.

The mean difference between bi-planar radiography and CR for minimum JSW of all compartments was 0.45 mm (9%, *p* < .001), likely attributed to the difference in leg positioning between modalities, including the left anterior foot offset and extended knee position used in the biplanar technique. Our findings imply that longitudinal monitoring of minimum JSW, and therefore serial evaluation of knee OA could by reproducibly assessed and feasible using either modality, but due to systemic differences in the absolute minimum JSW values, the modalities should not be intermixed. This is an important clinical and research consideration when developing an imaging protocol to assess patients or study participants for the assessment of knee OA.

As might be expected, leg positioning used for the bi-planar scans had a statistically significant effect of measured mean minimum JSW values. We found that the minimum JSW measurement of the forward unloaded left knee for both the medial and lateral tibiofemoral compartments was significantly greater than the back (right) knee. Comparison between modalities demonstrated that the bi-planar JSW of the forward (left) knee was greater than that measured using CR for both the medial (0.82 mm) and lateral (0.86 mm) tibiofemoral compartments. For context, these differences represented between 15.5% and 17.6% greater JSW measured on the forward knee with bi-planar radiography compared to CR. We note that the forward unloaded left leg is relatively “unloaded” compared to the back (right) leg in this position. Our findings are consistent with work by Kinds and colleagues who showed that conventional radiographs with the knee in extension led to a significant increase in minimum JSW measurements (+0.07 mm/cm of shift).^18^ Furthermore, work by Rueckl and colleagues highlighted that the PA semi-flexed (Rosenberg) view better classified the severity of lateral tibiofemoral OA when compared to the AP extended position in patients with valgus OA, and provides comparable diagnostic sensitivity for medial tibiofemoral JSW in varus OA.^19^ Although we could not eliminate the impact of the offset leg positioning used for biplanar imaging, the use of a positioning aid customized to each participant allowed maximum reproducibility of JSW measurements. If biplanar imaging with leg offset were to be used for longitudinal monitoring in studies or clinical management, it would be advisable to utilize a custom positioning aid for each participant to ensure reproducibility and maximize precision.

The offset foot positioning presumably results in a shift of the center of the subjects body mass such that the participant preferentially loads the forward leg less than the rear leg. Although this could be resolved by placing the feet next to each other during bi-planar radiography (equivalent to AP full length lower extremity radiographs), this would preclude simultaneously evaluating other alignment measures, such as femoral version. An obvious disadvantage of this approach is that it negates the benefit of using bi-planar radiography to simultaneously collect multiple alignment biomarkers (ie, unrelated to JSW) from a single scan. One approach that could be utilized to minimize the effects of weight-bearing on reproducibility is to use pressure sensors (ie, scales) under each foot, which would provide the participant immediate feedback to balance the weight-bearing load on each limb prior to image acquisition.

Although reproducibility was good for both modalities, there was a systematic difference in the JSW measures due to a combination of foot positioning and weight bearing. Knee flexion angle was shown by Guermazi and colleagues to significantly affect tibiofemoral JSW measurements based on knee radiographs at different degrees of joint flexion,^20^ likely because there is cartilage thickness variation across the knee joint. Semi-flexed weight-bearing views preferentially evaluate tibiofemoral JSW posterior of midline, where cartilage loss tends to be greater in OA. The screw home mechanism^21^ may play a role as well because it is sensitive to weight bearing (ie, forward left leg is unloaded), muscle activation, ligament tensioning, and the asymmetric articular surfaces. Compartmental differences also occur, as shown by Duddy and colleagues, who demonstrated that in a semi-flexed view compared to a standing extended view, the medial tibiofemoral compartment had significantly decreased JSW for the radiographic assessment of OA.^22^ While there are significant absolute differences in JSW between the biplanar and CR modalities, the reproducibility is similarly good, suggesting that either modality could be used to longitudinally assess changes in JSW within an individual.^23^ Future research could consider developing a correction factor to standardize the relationship between modalities. However, this would require a large cohort with a wide variation in JSW to be reliable.

Bi-planar radiography has some important advantages. For example, the radiation dose can be up to half of CR (although we did not use EOS-fast mode in our study to benefit maximally) and the total patient examination time is less. Furthermore, the collection of two simultaneous AP and LAT projections provides the potential for lower extremity alignment imaging biomarkers in addition to JSW to be obtained. Currently, the assessment of JSW is a manual analysis with a simplified outcome measure (ie, minimum JSW is a single measurement to represent a complex joint). However, the advantage of 2 simultaneous projections may allow future automation using a machine learning approach to provide a more comprehensive assessment of joint space metrics, including continuous JSW measures and joint congruency.

There are some limitations of our study to consider. Our study cohort was generally young, healthy participants, so it may not be representative of a cohort with knee OA. Although weight-bearing X-ray use is well-established in the setting of knee OA, the impact of the left foot forward, asymmetric weight-bearing position used for EOS in this study is unknown. The greater structural heterogeneity could potentially make JSW assessment more challenging. Furthermore, in an OA populations there may be difficulty maintaining a comfortable standing position that could also affect repeatability. Future studies would need to address these issues and their impact on the reproducibility of JSW measurements. We determined intra-rater repeatability, which was excellent, but ideally we would have also measured inter-rater repeatability. In clinical applications, it is likely that variation in the acquisition of biplanar scans would increase as the follow-up scans would be months apart, not minutes. However, this is not unlike conventional knee radiographs, where positioning devices are now utilized to reduce scan-to-scan variability. Another limitation of this study is that only the weight-bearing tunnel view was used to measure minimum JSW for comparison to bi-planar measures. Future studies might consider comparison to other routine knee radiographic views, such as the PA-semi-flexed Rosenberg view or AP extended view. However, adding that to our study protocol would have increased participant radiation exposure.

Although biplanar radiography was designed primarily for assessing joint alignment, our study demonstrates that it provides reproducible measurement of minimum tibiofemoral JSW in a healthy cohort and is a feasible modality that can be used to monitor knee health in patients at risk of developing OA. Presumably due to the offset foot forward positioning and asymentric distribution of subject weight, we demonstrated a systematic different and greater JSW measurement in the forward unloaded left leg tibiofemoral JSW compared to CR-based measures. With this in mind, whichever modality is used for prospective knee JSW evaluation, the modalities are not interchangeable. Differences in mean JSW measurement between biplanar imaging and conventional radiographic techniques might be reduced by careful standardization of foot positioning and ensuring even distribution of subject body weight between limbs. Maintaining the positioning with the foot offset allows measures in addition to JSW to be obtained simultaneously by biplanar imaging with EOS.

## Data Availability

Processed data will be made available upon reasonable request. Contact the corresponding author to request data.
